# Supplementary material for: Complementary benefits of multivariate and hierarchical models for identifying individual differences in cognitive control
Source: Imaging Neurosci (Camb). 2025 Feb 10;3:imag_a_00447. doi: 10.1162/imag_a_00447 (PMC11823007; doi:10.1162/imag_a_00447)
Supplement: Supplementary Material [file imag_a_00447-supp.pdf]

Supplementary Materials for Complementary benefits of multivariate and hierarchical models for identifying individual differences in cognitive control

Michael C. Freund*[1,3], Ruiqi Chen[2], Gang Chen[5], and Todd S. Braver[1,3,4]

[1]Department of Cognitive, Linguistic, and Psychological Sciences, Brown University
[2]Division of Biology and Biomedical Sciences, Washington University in St. Louis
[3]Department of Psychological & Brain Sciences, Washington University in St. Louis
[4]Department of Radiology, Washington University in St. Louis
[5]Scientific and Statistical Computing Core, NIMH, NIH, Bethesda, MD, USA

*Address correspondence to michael_freund@brown.edu

10 Supplementary Material

10.1 Validation of the timeseries modeling approach

To estimate single-trial BOLD responses, we used a simple method of selective averaging of responses from TRs at a fixed time-lag from each stimulus onset. Because this method can be inflexible and subject to substantial bias from adjacent trial's responses, we performed several validation analyses. These analyses confirmed that our design can support selective averaging, that the resulting estimates are straightforwardly interpretable as single-trial evoked responses, and are largely similar to those estimated from other popular estimation procedures.

First, we compare the TRs selected for averaging to aggregate estimates of the deconvolved event-related response contrast between incongruent and congruent trials, as estimated via an FIR model (9 left). This aggregate "Stroop-effect" timecourse (black) was averaged across all vertices within our ROIs, all sessions, and subjects (of a larger sample of N=80). We can see that the selected TRs (grey, vertical dotted lines) correspond to the "peak" of the Stroop-effect response. Additionally, we can see that these TRs correspond well to the peak of a fixed-shape HRF (red), obtained by convolving a 1-second boxcar with AFNI's *BLOCK* event model, selected based on prior work with this dataset (Freund et al., 2021). Therefore, this window captures the peak of the Stroop contrast well on aggregate, and approximates the time range that would be expected under typical HRF assumptions.

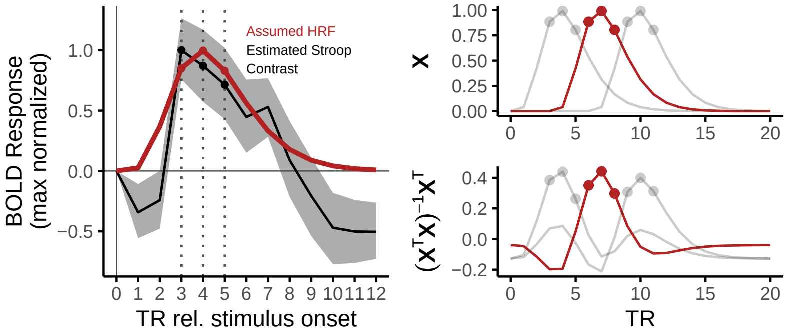

Figure 9: **Comparing selected averaging window to deconvolved Stroop contrast and an assumed HRF.**

Second, we examine via simple design matrix analysis, how using a convolution approach via regression might compare to use of selective averaging (Figure 9, right). Here, we assume a fixed-shape, 1 s HRF (AFNI's *BLOCK*). We create a design matrix consisting of an intercept and three separate predicted responses for three adjacent trials, separated in time by 3 intervening TRs (3.6 s), the minimum separation possible within our experiment (Figure 9, right top). We can see that a fair proportion of the middle trial's predicted response is non-overlapping with those from surrounding trials, and that this lack of overlap is particularly the case within the selected TRs. We see this more directly when computing the pseudoinverse of this design matrix, which illustrates the weights that the regressors assign to each TR (i.e., after accounting for the overlap; Figure 9, right bottom). The TRs that are weighted most strongly are those within the selected window. This suggests that, under the assumptions of this event model, selective averaging of this time window in our design should have a degree of robustness to collinearity. Indeed, when subjects' design matrices are built using separate *BLOCK* regressors per trial, a majority of trial regressors maintain small

variance inflation factors (VIF < 3).

Third, we conduct an empirical sensitivity analysis, in which we compare the estimates obtained from selective averaging to those obtained from two popular approaches for estimating single-trial responses, which we refer to here as "least-squares—separate" (LS-Separate) and "GLM-All with ARMA(1,1)." (see Mumford et al., 2012). These methods differ in several respects from selective averaging and so afford a strong test of sensitivity. LS-Separate is conceptually similar to selective averaging, as both permit correlation among adjacent trial responses, and hence a degree of estimation bias, with the goal of tempering estimation variance that arises from accounting for this collinearity (fitted via AFNI's *3dLSS*). GLM-All attempts to decorrelate all single-trial responses within a single model, and we fitted this within autoregressive GLMs (AFNI's *3dREMLfit*, which performs vertex-wise optimization of autoregressive parameters). We compared the selective averaging estimates to each of the other estimates in a variety of ways (Figure 10). In general, selective averaging yields estimates that are similar to both methods, but especially to the popular LS–separate method.

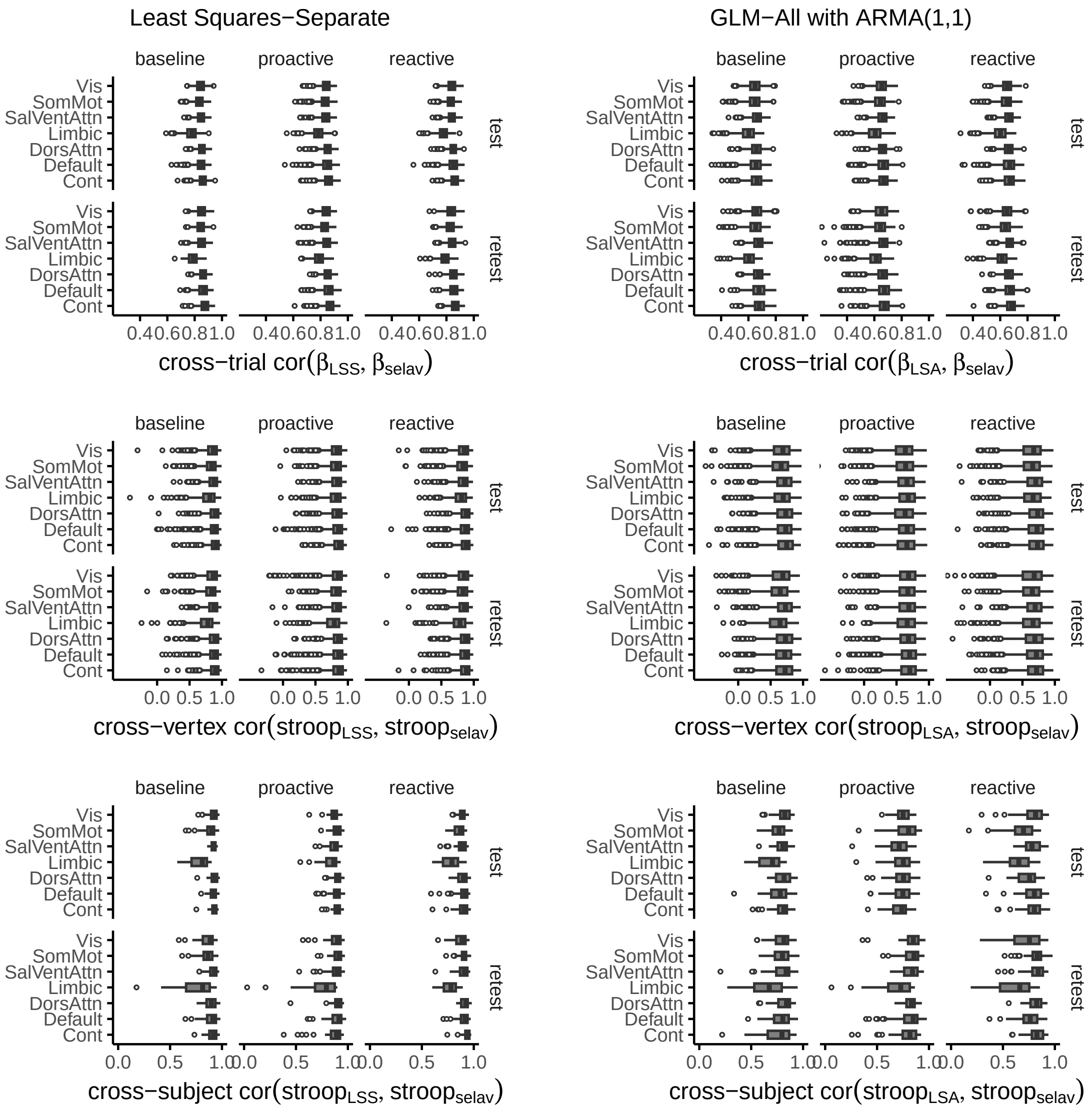

Figure 10: **Empirical sensitivity analysis** Estimates from selective averaging were compared to those from two other single-trial timeseries modeling methods: least-squares separate (**left**), and GLM-All with ARMA(1,1) (**right**). All boxplots summarize a distribution of correlations, and are organized by Schaefer-atlas Networks (**y-axes**). **Top**, Within each vertex, parcel, and subject, we estimated the correlation between models in their timeseries of single-trial estimates (i.e., with trials as observations). We then summarized these correlations within each parcel and subject by taking the median over vertices. Boxplots were drawn based on these median correlations (their distribution over parcels*subjects). **Middle**, Within each vertex, parcel, and subject, we estimated the mean Stroop contrast (mean of incongruent minus congruent trials). We then estimated the correlation between models in their spatial pattern of the Stroop contrast estimates (i.e., with vertices as observations). Boxplots were drawn based on these correlations (their distribution over parcels*subjects). (Figure 10, middle). **Bottom**, Within each vertex, parcel, and subject, we estimated the mean Stroop contrast, then took the mean contrast value over vertices. We then estimated the correlation between models in their univariate Stroop contrast estimates (i.e., subjects as observations), per parcel. Boxplots were drawn based on these correlations (their distribution over parcels). 10, bottom).

10.2 Model comparison

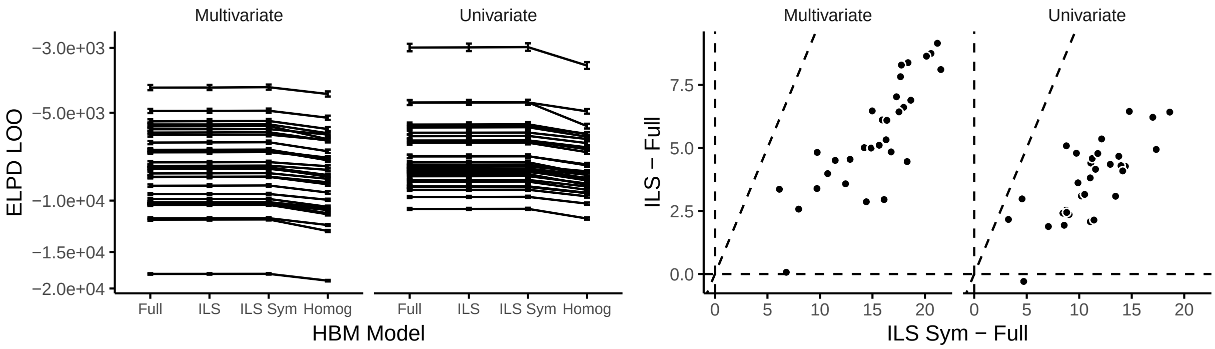

Figure 11: **Results of a model comparison.** This model comparison was conducted on 32 brain parcels (see Method). Expected-log pointwise predictable density (ELPD) was measured in a leave-one-out manner (LOO). **Left**, Lines connect ELPD LOO estimates (y axis) across different models (x axis) from the same parcel. More positive values indicate a better fit, in terms of better estimated ability of the model to account for out-of-sample datapoints. The pattern of ELPD LOO is highly consistent across parcels. Note that because statistics from the Homog. model were considerably lower (worse) than others, the y-axis spacing is non-linear (inverse hyperbolic sine function). **Right**, Within-parcel contrasts of ELPD LOO. Each point is a parcel. X and y axes illustrate the difference between ELPD LOO for the respective models. Dashed lines illustrate unity line and x and y intercepts. The pattern of ELPD LOO is highly consistent across parcels. On the x-axis, most parcels lie above 0, indicating ILS Sym was preferred over the Full model. On the y-axis, most parcels lie above 0, indicating ILS was preferred over the Full model. Additionally, all parcels lie to the right (underneath) the unity line, indicating ILS Sym was preferred over ILS.

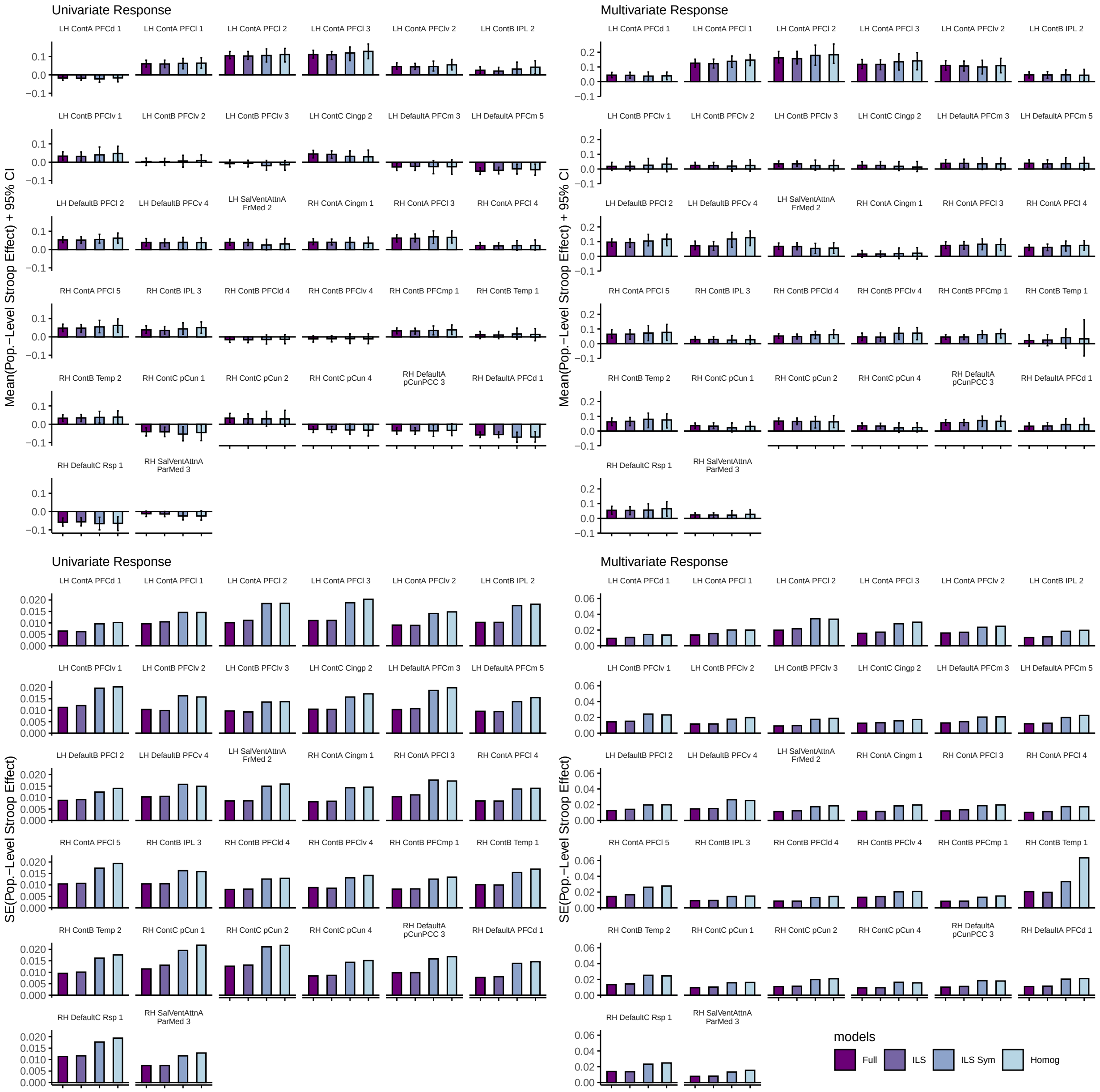

Figure 12: **Estimates of population-level Stroop contrasts for all reliability models fitted in 32 brain parcels used for model comparison. Top**, Bar heights illustrate the mean of the posterior, with errorbars illustrating 95% CI. **Bottom**, Bar heights illustrate the standard error in the mean, measured as the SD of the posterior distribution.

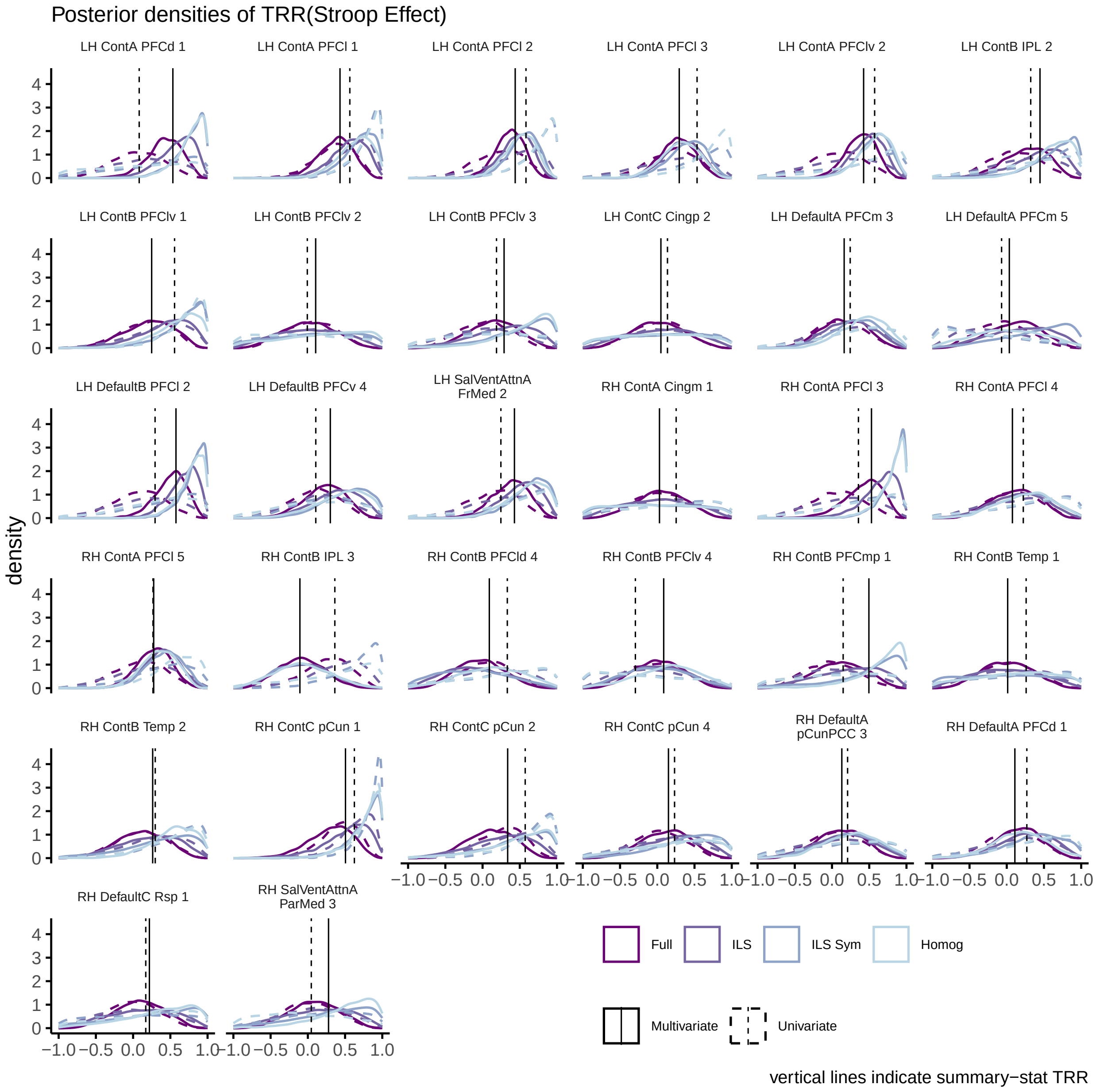

Figure 13: **Posterior densities for individual-level test–retest correlations for all reliability models fitted in 32 brain parcels used for model comparison.**

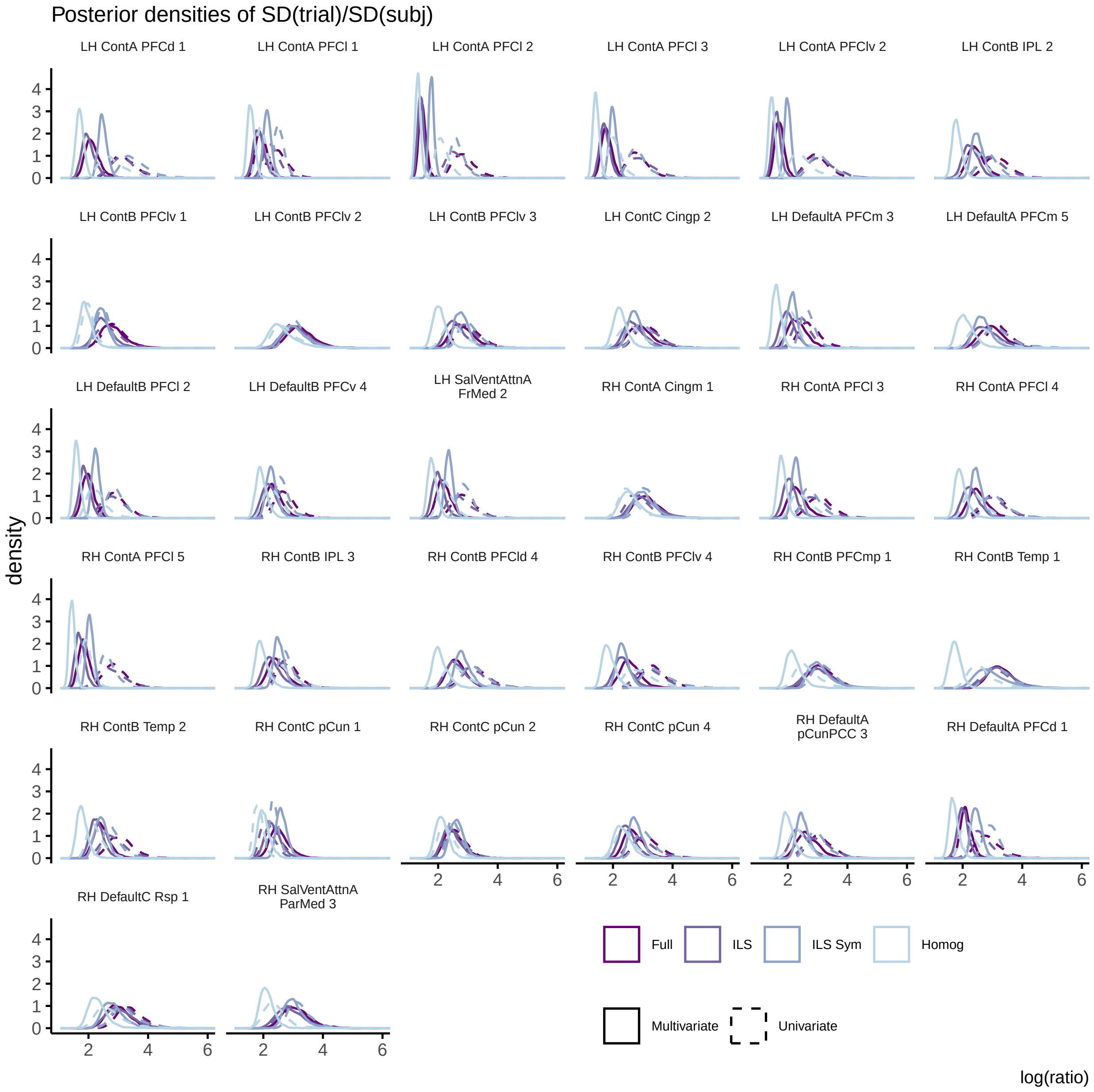

Figure 14: **Posterior densities for trial/subject variability ratios for all reliability models fitted in 32 brain parcels used for model comparison.**

10.3 Supplemental results from selected model (ILS Sym)

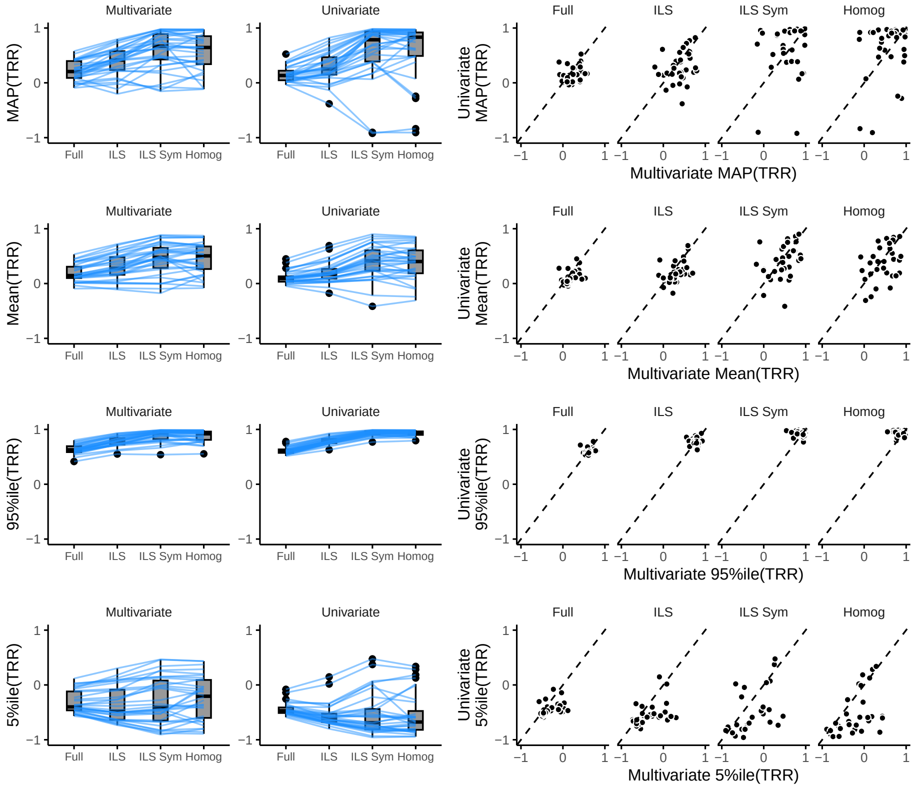

Figure 15: **Test–retest reliability statistics from all reliability models fitted in 32 brain parcels used for model comparison. Left**, On average across 32 *a priori* regions of interest and 5 models we fitted within a model-comparison analysis, reliability estimates tend to be positive, with many $r > 0.5$. Reliability estimates tend to shrink on average when more complex models are fitted (moving right to left on the x axis). As suggested by the model comparison statistics (11), the two most complex models (Full and ILS) are overfitted, while the least complex (Homog.) is underfitted, suggesting results from these models should not bear strongly on inferences. **Right,** Nevertheless, the impact of multivariate decoding relative to univariate contrasts can be seen clearly in the lower-bound estimate (5%-ile) of each model, as most lower-bounds are more positive with multivariate contrasts (x axis) than univariate contrasts (y axis; dashed lines display unity, and most points are below it).

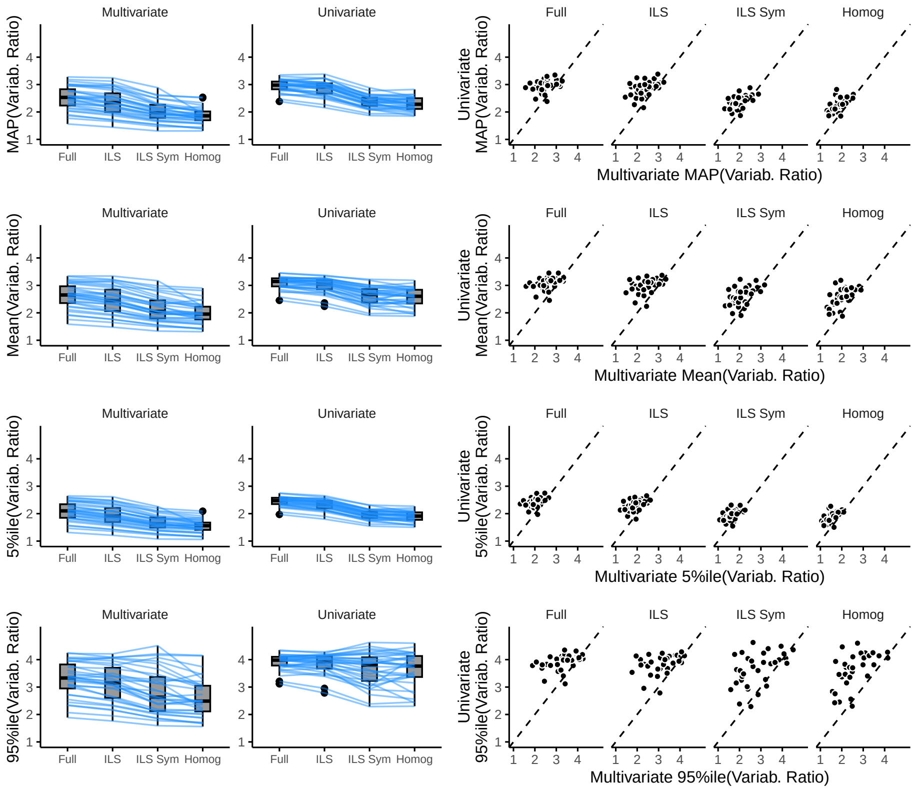

Figure 16: **Variability ratio statistics from all reliability models fitted in 32 brain parcels used for model comparison.** This figure is analogous to Figure 15. Variability ratio was defined as the log ratio of trial-level variability versus subject-level variability (see Method). Regardless of brain region or reliability model, variability ratios tend to be smaller (i.e., less relative trial-level variability) in multivariate contrasts as opposed to univariate contrasts.

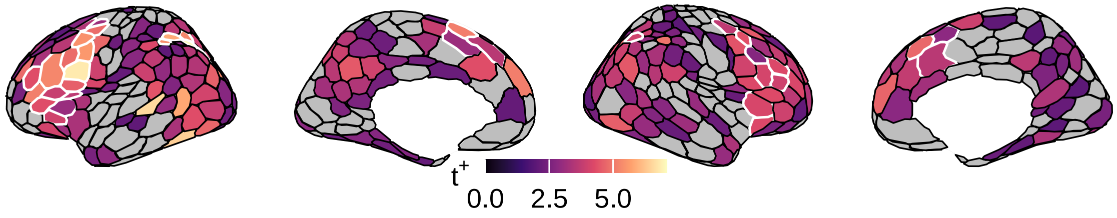

Figure 17: **Population-level multivariate contrast.** The hue of each parcel depicts the t^{+} statistic (see Method or Figure 2), computed on the multivariate Stroop contrast. Only parcels that contained highly discriminable incongruent vs congruent activation patterns are displayed in saturated hues, as defined by a > 0 threshold on the lower-bound of the 95% HDI.

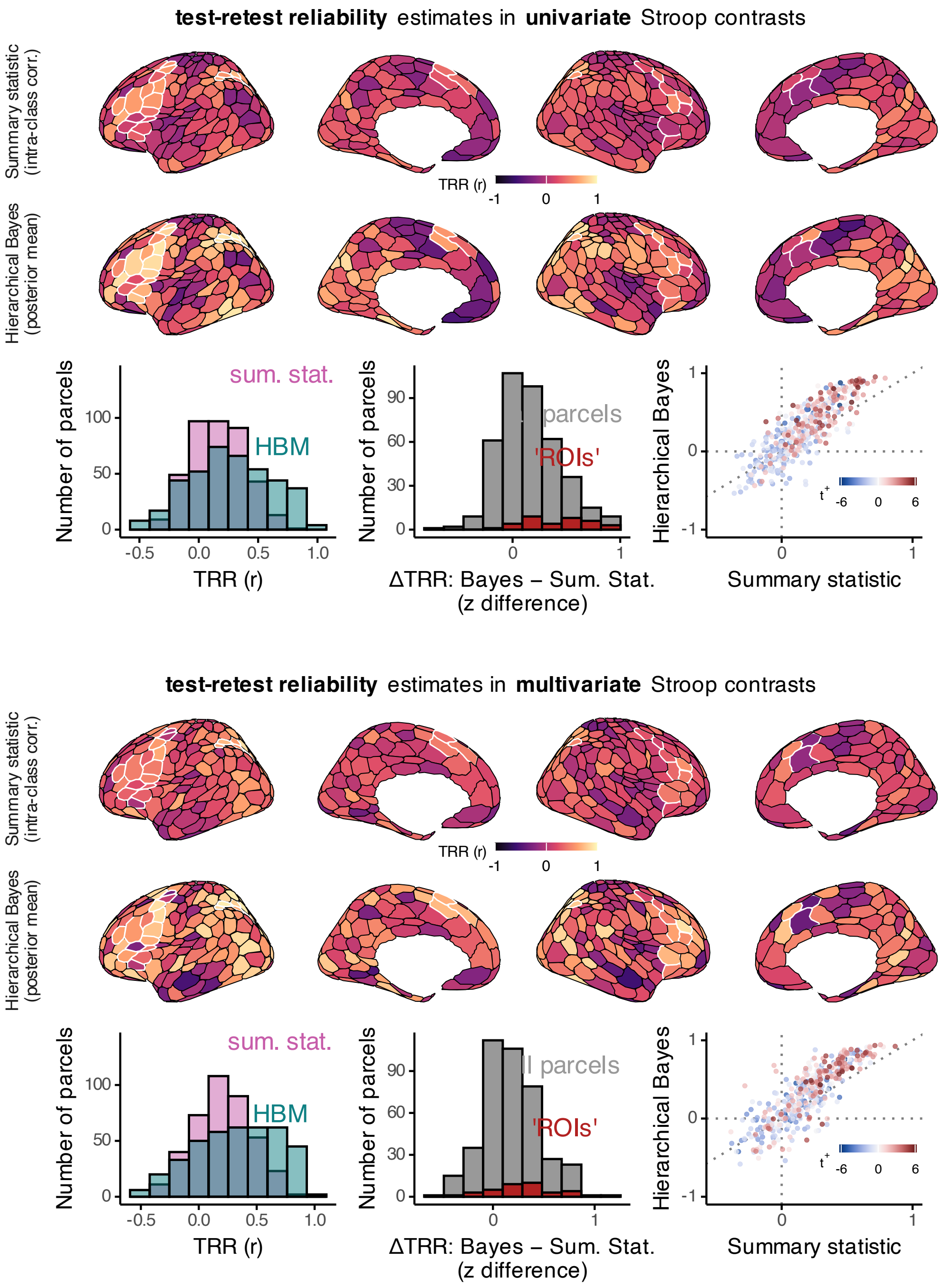

Figure 18: **Posterior mean test–retest reliability estimates in univariate (top) multivariate (bottom) Stroop contrasts.** All plots are analogous to those in Figures 3 and 4 but using mean rather than MAP.

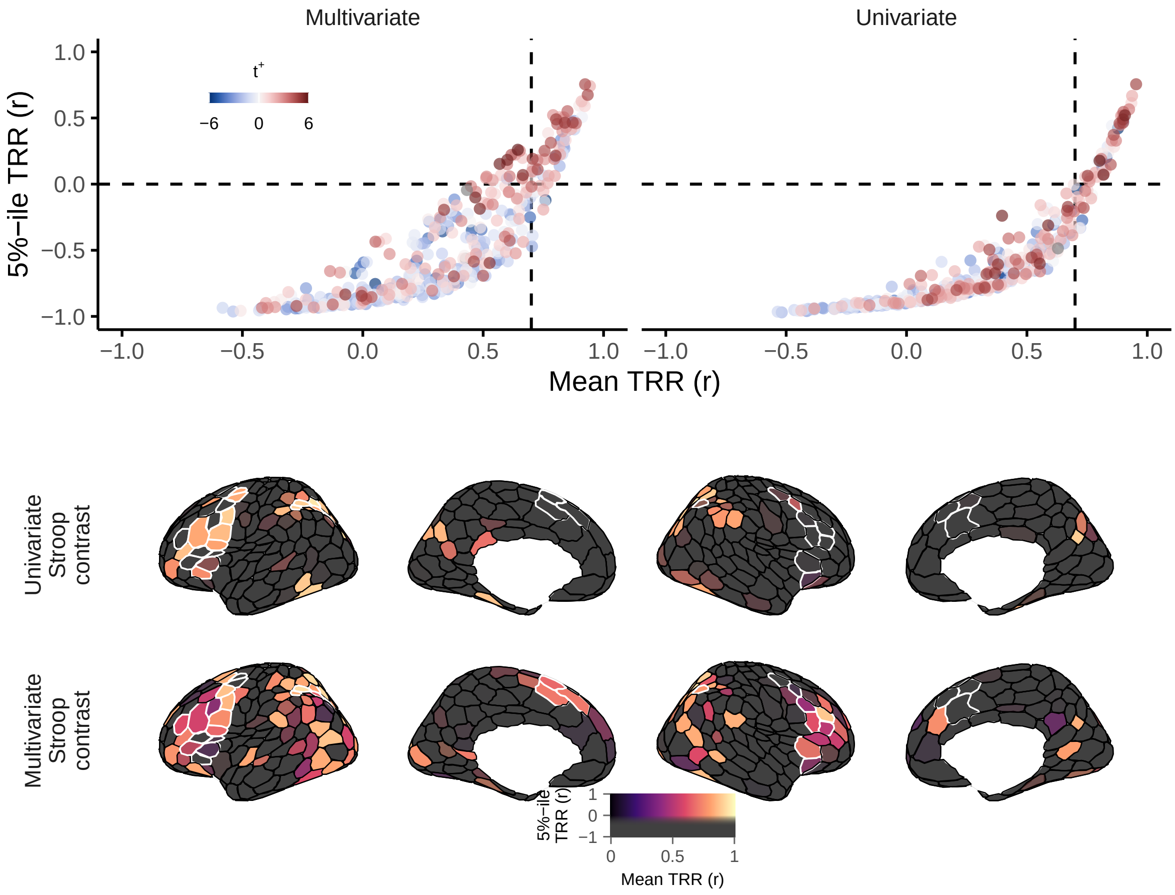

Figure 19: **Analogous to Figure 5, but with posterior mean TRR displayed instead.**

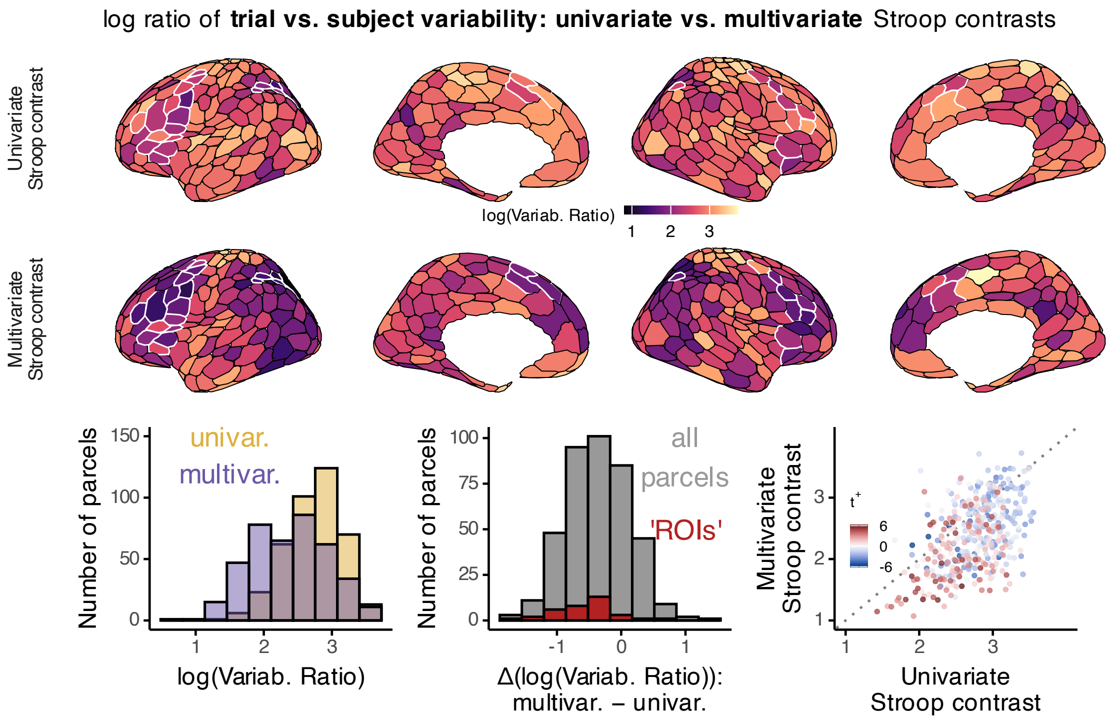

Figure 20: **Variability ratio in univariate versus multivariate contrasts.**

References

Freund, M. C., Bugg, J. M., & Braver, T. S. (2021). A Representational Similarity Analysis of Cognitive Control during Color-Word Stroop. *Journal of Neuroscience*, *41*(35), 7388–7402. https://doi.org/10.1523/JNEUROSCI.2956-20.2021

Mumford, J. A., Turner, B. O., Ashby, F. G., & Poldrack, R. A. (2012). Deconvolving BOLD activation in event-related designs for multivoxel pattern classification analyses. *NeuroImage*, *59*(3), 2636–2643. https://doi.org/10.1016/j.neuroimage.2011.08.076
